# The Effect of Extremely Low Frequency Alternating Magnetic Field on the Behavior of Animals in the Presence of the Geomagnetic Field

**DOI:** 10.1155/2015/423838

**Published:** 2015-12-28

**Authors:** Natalia A. Belova, Daniel Acosta-Avalos

**Affiliations:** ^1^Institute of Theoretical and Experimental Biophysics Russian Academy of Sciences, Institutskaya 3, Pushchino, Moscow 142290, Russia; ^2^Centro Brasileiro de Pesquisas Fisicas (CBPF), Rua Xavier Sigaud 150, Urca, 22290-180 Rio de Janeiro, RJ, Brazil

## Abstract

It is known that the geomagnetic field can influence animal migration and homing. The magnetic field detection by animals is known as magnetoreception and it is possible due to two different transduction mechanisms: the first one through magnetic nanoparticles able to respond to the geomagnetic field and the second one through chemical reactions influenced by magnetic fields. Another behavior is the magnetic alignment where animals align their bodies to the geomagnetic field. It has been observed that magnetic alignment of cattle can be disrupted near electric power lines around the world. Experimentally, it is known that alternating magnetic fields can influence living beings, but the exact mechanism is unknown. The parametric resonance model proposes a mechanism to explain that effect on living beings and establishes that, in the presence of a constant magnetic field, molecules associated with biochemical reactions inside cells can absorb resonantly alternating magnetic fields with specific frequencies. In the present paper, a review is made about animal magnetoreception and the effects of alternating magnetic fields in living beings. It is suggested how alternating magnetic fields can interfere in the magnetic alignment of animals and a general conclusion is obtained: alternating magnetic field pollution can affect the magnetic sensibility of animals.

## 1. Introduction

Living beings are sensitive to magnetic fields. For high intensity magnetic fields molecular diamagnetism becomes important and it is possible to observe levitation under the appropriate conditions [1]. It is interesting to ask about the interaction of living beings with magnetic fields whose intensities are similar to or lower than the geomagnetic field. In this case, living beings can detect (sense) static magnetic fields through specialized structures or organs, or they can be affected by temporal variations of those magnetic fields. Magnetic fields whose amplitude does not vary in time are called DC magnetic fields. Alternating magnetic fields (AMFs) are those fields whose amplitudes vary in time. The geomagnetic field (GMF) presents DC and alternating components. The sum of DC and alternating magnetic fields is called combined magnetic field (CMF). The aim of the present paper is to describe the before-mentioned situations for the geomagnetic field detection and for man-made alternating magnetic fields oscillating at frequencies lower than 100 Hz, considering magnetic field amplitudes in the range of *μ*T and lower, ending with a suggestion about the relation of both mechanisms in the animal magnetoreception process.

## 2. Geomagnetic Field

Living beings are born and grow under the presence of several physical fields, such as the gravitational and geomagnetic fields. In some way, living beings are affected by the physical characteristics of the GMF, because of their long time of relationship since the GMF is as ancient as the beginning of life [2]. The GMF vector can be characterized by three parameters: amplitude, inclination (relative to the vertical direction), and declination (relative to the geographical north-south axis). These values depend on the geographical coordinates and can be calculated using geomagnetic calculators such as the one available at the National Geophysical Data Center of NOAA-USA (http://www.ngdc.noaa.gov/geomag-web/#igrfwmm). It is not the aim of this paper to discuss the general characteristics of the GMF, since a plentiful literature of textbooks and review papers exists (e.g., [3]).

The GMF is generated mainly in the Earth's interior, and external magnetic fields generated in the magnetosphere and external space also contribute. The main contribution to the GMF can be understood as the result of a magnetic dipole in the Earth's interior, but the GMF is not fully dipolar. Some areas on the Earth's surfaces have anomalous GMF values, different from what is expected from a dipolar one [3]. In average, the GMF amplitude is about 50 *μ*T, and an example of anomaly is the South Atlantic geomagnetic anomaly (presently located in Brazil), where the GMF amplitude is the lowest in the world, being about 22 *μ*T [4].

In the temporal regime, the GMF parameters present changes over great time periods (about hundreds to thousands of years). These variations are known as the secular variations [3]. The GMF also presents fast variations during geomagnetic storms with typical frequencies from 0.001 Hz to 10 Hz [5] and daily variations with a 12-hour period that corresponds to the hours of daylight [6]. It is acceptable to assume that, for evolutionary reasons, living beings are insensible to secular variations, because these periods are greater than the maximum life span of any living being. On the other hand, living beings must be sensible to the fast and daily variations.

Added to the geomagnetic field are the man-made magnetic fields, arising from the electric power lines, house electrical circuits, and appliances conducting alternating electrical currents at 50 or 60 Hz, producing AMFs with amplitudes in the order of hundreds of nT. These fields have a period of about 17 ms, very short when compared with the natural variation in the GMF (12 hs for daily variation and about minutes or hours for geomagnetic storms). But even so, experimental observations have shown that AMFs at very low frequencies can alter animal behavior [7–10].

## 3. Detection of the GMF

### 3.1. Experimental Evidence

The first evidence that living beings can be influenced by the GMF was the discovery of magnetotactic bacteria. They were observed first by Salvatore Bellini in 1963 [11, 12] and later by Blakemore in 1975 [13]. Magnetotactic bacteria are microorganisms with the ability of aligning their swimming direction to the geomagnetic field lines, ability known as magnetotaxis. To accomplish that, they biomineralize magnetic minerals in organelles known as magnetosomes [14]. The typical magnetic minerals found in magnetotactic bacteria are nanoparticles (average size from 50 to 100 nm) of magnetite (Fe_3_O_4_) or greigite (Fe_3_S_4_), typically in geometrical cubo-octahedron, cubes, or even bullet-shaped forms [14]. Magnetosomes are organized in the cytoplasm in chains. These chains confer to the bacteria a magnetic moment that permits the orientation of their navigation. These bacteria can be found as coccus, vibrions, spirilla, or even multicellular forms known as multicellular magnetotactic prokaryotes [15]. They are found in aqueous sediments such as marine ambient, rivers, and lakes. It is believed that magnetotactic bacteria use their magnetic ability to get to deeper sediments easily, in places where the oxygen concentration is optimum for them [16].

The magnetic nanoparticles in magnetosome chains are characterized as single domains, meaning that the magnetic moment is stable in time and under temperature changes, different from superparamagnetic particles [17]. In magnetotactic bacterial populations, exposure to oscillating strong magnetic fields (more than 10^5^ *μ*T, 50 or 60 Hz) provokes the inversion of the magnetic moment direction at about 50% of the population, agreeing with the idea that magnetosomes are single domains [18]. Observation of the interaction of magnetotactic bacteria with the GMF through magnetic nanoparticles encouraged the idea that animals should detect the GMF using a similar mechanism. In animals, the use of vectorial information from the GMF in orientation and navigation tasks is well documented, an ability known as magnetoreception [19]. Several laboratory experiments have shown that social insects, such as bees and ants, can use magnetic field information in orientation tasks [20]. In migratory birds, the choice of flight direction is influenced by the local GMF [21]. Another phenomenon that recently gained new interest is magnetic alignment [22], which is related to the alignment of the body axis to the GMF lines or to the GMF horizontal component. Firstly, it was identified in termites, bees, and fruit flies [19]. But it attracted attention when the same behavior was identified in cows and deer [23]. Also the orientation of the body axis to the GMF lines in carp can be observed in still water in tanks [24].

The analysis of migration and homing in animals and their correlation with different magnetic stimuli permit the identification of two magnetic orientation mechanisms [25]:

- Polarity compass: in this case the animal can sense the GMF horizontal component, like a compass, to elucidate the magnetic north direction and use this information in orientation tasks, most likely to be used by animals in foraging and short distance migrations.
- Inclination compass: the animal senses the GMF vertical component. This sense permits the animal to identify the Earth hemisphere and the direction to the geomagnetic equator, most likely to be used by animals in long distance migrations.

Experiments done with turtles and birds [26] showed that in some way animals use the geomagnetic field parameters to know their geographical position on the Earth. To explain that behavior, the animal magnetic map model has been proposed. The exact parameters used in this map are not known, and it is speculated that they can be the geomagnetic inclination and intensity [27].

It has been observed in different bird species and other animals that in some cases magnetoreception just happens in the presence of light, a phenomenon known as light-dependent magnetoreception [28, 29]. This kind of magnetic detection depends also on the wavelength, being observed in birds, an effective orientation for short wavelengths (<500 nm) and disorientation for long wavelengths (>500 nm) [30]. In some cases the light-dependent magnetoreceptor is in the eyes and, in some birds, there is laterality in the eye with magnetoreception function [31, 32]. In other animals, the magnetoreception is extraocular [33].

### 3.2. GMF Transduction

Up to the present, magnetoreception is understood to be due to two possible mechanisms: transduction through magnetic nanoparticles or transduction through light-depending chemical reactions involving radical intermediates [34].

The transduction through magnetic nanoparticles, also known as the ferromagnetic hypothesis, was inspired by the existence of magnetotactic bacteria. This assumes that there must exist a specialized organ or structure able to detect magnetic fields [35]. Inside this organ must be magnetic nanoparticles forming chains or another kind of structure able to generate mechanical torques or other dynamic effects in the presence of magnetic fields. The interaction of these magnetic nanoparticles with the magnetic field must produce a corresponding cellular signal, for example, through the mechanotransduction of magnetic torque. As the magnetic properties of magnetic nanoparticles depend on the size, different possibilities have been proposed for superparamagnetic and for single domain nanoparticles. However, in all cases the important point is that the generated torques and strains must be mechanically transduced [36]. The ferromagnetic hypothesis can be tested by measuring the magnetization of, or isolating magnetic nanoparticles from, body parts where the magnetic sensor is expected to be. Magnetic nanoparticles have been isolated from ant heads and antennae [37], termite bodies [38], trout noses [39, 40], and birds upper beaks [41], among others. Magnetization has been measured in insects [20] and lateral line in fishes [42–44], among others. In all these cases there is a correlation between the presence of magnetic material and magnetoreception in the animal, strengthening the ferromagnetic hypothesis.

In the case of light-dependent magnetoreception, it is accepted that the mechanism is related to light sensitive chemical reactions involving radical intermediates [45]. This mechanism is known as radical pair mechanism (RPM). Several evidences suggest that the target molecule for RPM is cryptochrome, present from bacteria to human beings [46]. The light-dependent chemical reaction produces a radical pair in a singlet state from ground state precursors, and the presence of magnetic fields converts a proportion of radical pairs from singlet state to triplet state. These radical pairs in singlet or triplet state react producing singlet products or triplet products at different rates. In some way, the modification in the rate of production of these products modifies the way the animal sees the world, allowing it to define a magnetic visual reference [47]. Of course, the last mechanism is not general, because in newts the light-dependent magnetoreceptor is not in the eyes [48].

## 4. Magnetic Alignment Can Be Affected by AMF of 50/60 Hz

As mentioned above, it has been shown that several animals can align their body axis with the GMF axis, the phenomenon known as magnetic alignment [19, 22]. Burda et al. [9] showed that the magnetic alignment of cows and deer can be disrupted near high-voltage power lines in the field. Interestingly, for power lines oriented east-west, generating AMF oriented north-south, they observed that cows aligned their bodies preferentially in an axis shifted about 90° with respect to the GMF axis. For power lines oriented north-south, generating AMF oriented east-west, cows oriented randomly. When cows were observed in different distances from the power lines, they return to align their bodies to the GMF axis at about 150 m of distance. The 90° shift for AMF oriented north-south is intriguing. Two interpretations are possible: cows orient following the power line orientation or the interaction between the GMF and the AMF produces that shift. The first hypothesis can be ruled out because for power lines oriented north-south cows became disoriented, not following the power line orientation. On the other hand, these two observations can lead us to conclude that two different mechanisms are involved in the disorientation of cows with AMF: one related to the interaction of GMF and AMF when they are parallel and the other related to the interaction when they are perpendicular. In the following sections, the experimental evidence of AMF effects on biological systems and one model that explains these effects assuming an interaction between parallel static and alternating magnetic fields are shown.

## 5. Effects of AMFs: Models and Experimental Evidence

There are reports indicating that extremely weak AMFs (EW AMF) with values of magnetic field amplitudes in *μ*T, nT, and even pT ranges are able to induce statistically significant effects in biological systems. It should be noted that in most cases the experiments with EW AMF are performed in the presence of the static GMF. Moreover, it is possible that the presence of a DC magnetic field (MF) should be necessary for the induction of effects of AMFs. In general, the observed effects are the result of exposure of the biological systems to AMF or to combined AC and DC MFs (CMF), wherein the DC and AC components may be oriented relative to each other arbitrarily.

### 5.1. Biological Effects of the EW AMF of Power Frequencies

The possibility of the induction of biological effects of EW AMF is of particular interest to researchers for several reasons. One of them is that the AMF of anthropogenic origin is considered as a potential threat to human health [49]. Currently, there is a worldwide debate about the health risks due to exposure to low frequency electromagnetic fields. Several studies described adverse effects related to these fields while others observed no interactions with biological systems [50–52].

The available theoretical and experimental data indicate that AMF with a frequency of 50/60 Hz can induce biological effects in terms of MF amplitudes greater than 10 *μ*T, while the possibility of biological effects of AMF of amplitudes less than 10 *μ*T is questionable [53, 54]. However, several experimental works have shown the effect of EW AMF on biological systems. Among the experimental studies, the works of Liburdy et al. should be noted, which demonstrated the ability to block the inhibitory effect of physiological concentrations of melatonin and tamoxifen on the growth of human breast cancer cells (MSF-7) in culture, when they are exposed to sinusoidal MF with a frequency of 60 Hz in the *μ*T range [55–57]. Importantly, the results of Liburdy et al. [55] were confirmed independently in two laboratories [58, 59]. Ishido et al. [60] confirmed the Liburdy experiments using EW AMF of 50 Hz. Liburdy et al. [55] revealed the existence of a threshold value of the field amplitude (0.5–1.7 *μ*T) at which the bioeffect starts to be seen. Another study was devoted to the influence of sinusoidal 60 Hz AMF with amplitude ranging from 1 *μ*T to 20 *μ*T on the enzymatic activity of ornithine decarboxylase (ODC) in cultured fibroblast cells [61]. They observed the enhancement of ODC activity induced by the exposure of culture cells to the AMF suggesting a sigmoidal relationship to the MF amplitude, and an approximate doubling of the ODC activity was observed at field amplitude of 7 *μ*T or higher.

The biological effectiveness of MF of about 1 *μ*T has been shown in different test systems and using various combinations of frequency and AC amplitude. Fitzsimmons et al. [62] observed an increase in mitochondrial activity in cell culture spine HBV 155 using sinusoidal MF (*B*_AC_ = 0.8 *μ*T, *f* = 18 Hz). Lednev and Malyshev [63] showed that a sinusoidal magnetic field (*B*_AC_ = 1.0 *μ*T, *f* = 35.8 Hz) inhibited the Mg^2+^-ATPase activity of actomyosin in a cell-free system. In a series of works, Temuryants et al. showed the effect of a weak AMF at 8 Hz and amplitude of 5 *μ*T on several physiological and biochemical parameters in rats with hypokinesia. In particular, they showed that, under these conditions, EW AMF corrects lipid metabolism [64], corrects the phagocytic activity of neutrophils [65], and changes the temporal organization of physiological processes [66, 67].

The results of experiments using sinusoidal EW AMF support the conclusion that there is biological activity of AMF at about 1–10 *μ*T. It is known that the amplitudes of the power-frequency magnetic fields (50 or 60 Hz) in most of the living places range from 0.01 to 1-2 *μ*T, while at some workplaces it may reach 5-6 *μ*T [68]. However, questions about the mechanism of action of these fields remain open.

### 5.2. EW AMF and Geomagnetic Pulsations and Storms

Some publications show correlations among various medical or biological parameters and geomagnetic disturbances appearing during magnetic storms.

The geomagnetic field presents pulsations with periods from 0.2 s to 600 s, corresponding to a frequency band from 0.001 Hz to 5 Hz. This geomagnetic pulsation is called Pc or pulsation continuous [68]. There is the assumption that the Pc1 frequency band (0.5–2.0 Hz) coincides with the basic rhythms of the heart and the Pc3 pulsations, with periods from 20 s to 40 s (such quasi-periods were also seen in heart rhythm), could be biotropic agents of magnetic storms [69].

Long-term observation studies have shown a correlation between the number of emergency calls due to myocardial infarction, hypertensive crisis, and the mortality of people with cardiovascular diseases and the total duration of the Pc magnetic pulsations with a frequency of 0.2 to 5.0 Hz and amplitudes in the range of tens to hundreds of pT [70–74].

Experimental studies in rabbits have shown that exposure to magnetic storms leads to significant changes in the morphological and functional state of the heart and systems associated with its activities [75]. In studies with the stingless bees* Schwarziana quadripunctata* it was observed that the nest-exiting flight direction changes significantly during geomagnetic storms (amplitude variations of about 50 nT) [76]. The same behavior was observed in the stingless bee* Tetragonisca angustula* when the geomagnetic storm was simulated in the field [77]. Krylov et al. [78] showed the influence of H-components of a typical magnetic storm modeled in the laboratory on the early evolution of a* Daphnia magna*. Assessment rates of early ontogenesis of* Daphnia* showed that the effect of a magnetic storm since the outbreak in early ontogeny leads to changes in the size of the offspring in the first broods.

### 5.3. Lednev's Model on the Influence of EW AMF

Earlier, the Russian physicist V. V. Lednev, based on the ion cyclotron resonance model [79], proposed the parametric resonance model where it is considered that ions bonded to proteins (Ca^2+^, K^+^, and/or Mg^2+^) behave as isotropic coupled oscillators. These ions can serve as primary targets for CMF [80–82]. Considering a CMF as the sum of parallel AC and DC fields, the field can be written as *B* = *B*_DC_ + *B*_AC_ · cos⁡(2*πft*). Lednev's theory shows that the probability of biological effects by CMFs is described by the square of the Bessel function of the first order: *p* = *J*_1_^2^(*B*_AC_/*B*_DC_). Accordingly, the resonance frequency formally corresponds to the cyclotron frequency [79] *f*_*c*_ = *qB*_DC_/(2*πm*), where *q* is the ionic charge and *m* is the ion mass, and the maxima effects are achieved when *B*_AC_/*B*_DC_ = 1.8. The same mathematical prediction can be obtained using a different theoretical approach: the analysis of the velocity of the damped ion under the influence of the Lorentz force [83]. In both cases, the prediction of a dependence on specific values for *B*_AC_/*B*_DC_ has been tested in several experiments [84]. For the case of weak *B*_AC_ (less than 10 *μ*T), it has been shown experimentally that Lednev's model can describe the biological effects (amplitude and frequency dependences) of the CMF tuned to the Larmor precession frequency for some nuclear spins as ^1^H, ^39^K, ^55^Mn, ^31^P, ^35^Cl, ^63^Cu, and ^23^Na [85, 86]. This model permits the calculation of the AMF parameters necessary, on one hand, for achieving a maximal effect and, on the other hand, at known experimental AMF parameters, for the identification of primary targets [84, 87]. An experimental confirmation of this assumption in Lednev's model is provided by the results presented in [88–90], using two test systems: regenerating planarians and gravitropic reaction of plants. The results of Belova et al. [89] suggest that for fields of industrial frequencies (50 and 60 Hz) the primary targets are the spins of nuclei of hydrogen atoms.

## 6. The Disturbance of the GMF Detection by AMF

As mentioned above, it has been observed that 50/60 Hz AMF can disturb magnetic alignment [9]. These results are intriguing and show an interesting relationship: for AMF parallel to the GMF, animals show a shift of 90° in the alignment, and for AMF perpendicular to the GMF, animals became disoriented. These observations seem to be related to two different mechanisms. The first one fulfills one premise for Lednev's model (AMF parallel to GMF). The second one can be explained assuming that magnetic nanoparticles in clusters of superparamagnetic particles or interacting multidomain iron-mineral platelets involved in the GMF detection, or even radical pair reactions, are disturbed by the AMF as shown by Vanderstraeten and Gillis [91]. Vanderstraeten and Burda [92] discuss this phenomenon and propose that the magnetosensory disruption caused by the low frequency AMF must be analyzed rather than the accurate sensing of AMF. They disconsider the fact that for AMF parallel to the GMF animals in fact are oriented [9]. For this situation perhaps the effect is not on the magnetosensor but in the following steps to the MF transduction. As was mentioned above, Lednev's model considers that bonded ions associated with fundamental cellular functions can absorb resonantly the AMFs tuned to the cyclotron frequency of the ion, disrupting or enhancing its cellular function. As the models to explain magnetoreception through magnetic nanoparticles assume that these particles are inside special organelles, perhaps the ions related to the transduction of the magnetic torque can absorb AMFs resonantly at their own cyclotron frequencies, changing the animal MF perception. In some mechanotransduction systems, the stress produces a cellular Ca^2+^ influx [93], this ion being considered the principal target for CMF's biological effects. So, low frequency AMFs could disturb some steps in the GMF transduction process. Even in the case of cryptochrome associated with the radical pair mechanism, its signal transduction can be related to Ca^2+^ influx in some cases [94], being possible that low frequency AMFs disturb light-dependent magnetoreception. We recommend that future experiments addressing the relation among magnetoreception and 50/60 Hz AMF should be done considering the resonant absorption of Ca^2+^ or other ions related to the magnetic signal transduction.

The present time is characterized by great technological advances that bring together electromagnetic pollution. Power lines and mobile transmission antennas are sources of this pollution but in different frequencies. Recently, it has been shown that electromagnetic noise, in the frequency range of 50 kHz to 5 MHz, can affect the magnetic compass orientation of migratory birds, becoming totally disoriented [95]. The results of Burda et al. [9] show a similar result for extremely low frequencies in mammals. A general conclusion from both studies is that alternating magnetic field pollution in higher and lower frequencies can affect the magnetic sensibility of animals, and animal preservation policies must be aware of this.
